# A Small Peptide Modeled after the NRAGE Repeat Domain Inhibits XIAP-TAB1-TAK1 Signaling for NF-κB Activation and Apoptosis in P19 Cells

**DOI:** 10.1371/journal.pone.0020659

**Published:** 2011-07-18

**Authors:** Jennifer A. Rochira, Nicholas N. Matluk, Tamara L. Adams, Aldona A. Karaczyn, Leif Oxburgh, Samuel T. Hess, Joseph M. Verdi

**Affiliations:** 1 IGERT Functional Genomics Ph.D. Program, University of Maine, Orono, Maine, United States of America; 2 Center for Molecular Medicine, Maine Medical Center Research Institute, Scarborough, Maine, United States of America; 3 Department of Physics and Astronomy, University of Maine, Orono, Maine, United States of America; 4 Graduate School of Biomedical Sciences, University of Maine, Orono, Maine, United States of America; University of Texas MD Anderson Cancer Center, United States of America

## Abstract

In normal growth and development, apoptosis is necessary to shape the central nervous system and to eliminate excess neurons which are not required for innervation. In some diseases, however, apoptosis can be either overactive as in some neurodegenerative disorders or severely attenuated as in the spread of certain cancers. Bone morphogenetic proteins (BMPs) transmit signals for regulating cell growth, differentiation, and apoptosis. Responding to BMP receptors stimulated from BMP ligands, neurotrophin receptor-mediated MAGE homolog (NRAGE) binds and functions with the XIAP-TAK1-TAB1 complex to activate p38^MAPK^ and induces apoptosis in cortical neural progenitors. NRAGE contains a unique repeat domain that is only found in human, mouse, and rat homologs that we theorize is pivotal in its BMP MAPK role. Previously, we showed that deletion of the repeat domain inhibits apoptosis, p38^MAPK^ phosphorylation, and caspase-3 cleavage in P19 neural progenitor cells. We also showed that the XIAP-TAB1-TAK1 complex is dependent on NRAGE for IKK-α/β phosphorylation and NF-κB activation. XIAP is a major inhibitor of caspases, the main executioners of apoptosis. Although it has been shown previously that NRAGE binds to the RING domain of XIAP, it has not been determined which NRAGE domain binds to XIAP. Here, we used fluorescence resonance energy transfer (FRET) to determine that there is a strong likelihood of a direct interaction between NRAGE and XIAP occurring at NRAGE's unique repeat domain which we also attribute to be the domain responsible for downstream signaling of NF-κB and activating IKK subunits. From these results, we designed a small peptide modeled after the NRAGE repeat domain which we have determined inhibits NF-κB activation and apoptosis in P19 cells. These intriguing results illustrate that the paradigm of the NRAGE repeat domain may hold promising therapeutic strategies in developing pharmaceutical solutions for combating harmful diseases involving excessive downstream BMP signaling, including apoptosis.

## Introduction

Apoptosis is an evolutionarily conserved mode of programmed cell death and is necessary for multicellular organism development and cellular homeostasis. It is mediated by two central death pathways: the extrinsic pathway, which uses cell surface death receptors; and the intrinsic pathway, involving mitochondria and endoplasmic reticulum [1]. Both pathways utilize caspases which are cleaved from their inactive form by initiator caspases to become executioners of apoptosis targeting substrates for proteolysis and leading to the dismantling of cells. p38^MAPK^ is a mitogen-activated protein kinase that responds to extracellular stimuli (mitogens) to transduce signals from the cell membrane to the nucleus for inflammation, cell growth, differentiation, and apoptosis depending on the stimulus and the stress induced on the cells. One such stimulus is a group of growth factors and cytokines known as bone morphogenetic proteins (BMPs) which were originally identified in their role to induce the formation of bone and cartilage [2] but have also been found to be instrumental in the differentiation of nerve cells [3], dorsal-ventral patterning [4], and apoptosis [5].

BMPs are a large subgroup in the TGF-β superfamily. Like other members of the TGF-β family, BMP signaling is mediated through the activation of BMP serine/threonine kinase type I and II receptors by BMP ligands and activation of the SMAD proteins. [6]. However, studies have confirmed that BMPs can also directly activate the MAPK pathway [7], [8] depending on the order of assembly of the receptors [9]. TGF-β activated kinase 1 (TAK1) was originally identified as a mediator for BMP and TGF-β in the MAPK signal transduction pathway [10]. TAB1 (for TAK1 activating binding protein) activates TAK1 in BMP signal transduction [11], [12] and binds to the BMP receptors through X-linked inhibitor of apoptosis protein (XIAP) [10]. XIAP contains a RING zinc finger domain that interacts with the BMP receptors and three baculoviral IAP repeat (BIR) domains that bind with caspases to hinder apoptosis [13], [14]. TAK1 activates p38^MAPK^ [15], Jun N-terminal kinases (JNKs), and NF-κB [16].

We first identified NRAGE as a binding partner to the intracellular domain of p75 neurotrophin receptor (p75^NTR^) facilitating apoptosis in sympathetic neural progenitors [17]. NRAGE has been further investigated for its role in neural apoptosis [17], [18], [19], [20], [21], cell cycle regulation [22], cell-cell adhesion [23], melanoma and pancreatic cancer metastasis suppression [24], and renal branching morphogenesis [25]. NRAGE is a member of the MAGE family, which was originally identified in a screen for antigens expressed on the surface of tumor cells. Although NRAGE is expressed by tumors, it does not code for tumor antigens, unlike most MAGE members, is expressed in most developing and adult tissues. NRAGE contains a unique domain of 25 consecutive hexapeptide repeats with a consensus sequence of tryptophan-glutamine-x-proline-x-x (WQxPxx, where x is any amino acid) that we hypothesize is instrumental in its function. NRAGE binds with the XIAP-TAB1-TAK1 complex in the BMP MAPK pathway and aids in the activation of p38^MAPK^ and caspase-3. Furthermore, disruption of NRAGE in this pathway is sufficient to block phosphorylated p38^MAPK^ activation in mouse cortical neural progenitors and P19 embryonal carcinoma cells [26]. We have also shown that the XIAP-TAB1-TAK1 complex requires NRAGE for IKK-α/β phosphorylation and NF-κB activation [27]. Recently, we constructed a series of NRAGE deletion mutations and determined that the repeat and MAGE2 homology domains are responsible for activating p38^MAPK^ and caspase-3 [28]. Jordan et al. have determined that NRAGE co-precipitates with the RING zinc finger domain of XIAP [29]. However, the portion of NRAGE that interacts with XIAP has not been identified. Here, we used Förster-type resonance energy transfer (FRET) analyses to reveal that the interaction between NRAGE and XIAP is not only likely to be direct, but that the interaction is at the repeat domain in NRAGE. Furthermore, the repeat domain is required for IKK phosphorylation and NF-κB translocation. Thus the repeat domain is required for caspase, p38^MAPK^, and NF-κB activation, and serves as an intriguing cell signal switch with therapeutic potential. To test the possibility of targeting the NRAGE repeat domain for therapeutic purposes, we designed a small peptide mimicking the NRAGE repeat domain and found that it can inhibits binding of Xiap/Tak/TAb and reduces apoptosis in P19 cells. Because development and some diseases have a number of overlapping pathways including the BMP MAPK pathway, it is relevant that an investigation be conducted to devise potential therapeutic options that require a reduction in NF-κB activation as well as BMP-mediated XIAP-TAB-TAK1 signal transduction such as neurodegenerative, cardiovascular, and autoimmune disorders.

## Results

### Endogenous NRAGE and XIAP protein expression co-localizes primarily in the cytoplasmic compartment when imaged by confocal microscopy

**Figure 1:**
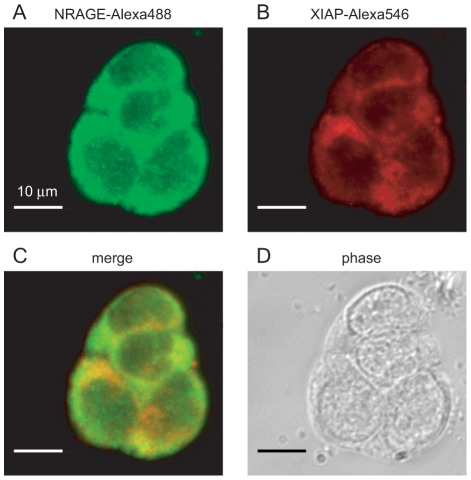
Endogenous NRAGE and XIAP expression in P19 cells. P19 cells were fixed with 4% PFA and permeabilized followed by application of primary antibodies for NRAGE and XIAP and widefield imaging. Secondary Alexa 488 and 546 antibodies were used to identify NRAGE (A) and XIAP (B) respectively. (C) Merged imaging shows NRAGE and XIAP mainly occupy the cytoplasm with smaller concentrations in the nucleus. (D) Phase contrast image.

Since caspase activation is a downstream effect of phosphorylated p38^MAPK^ and XIAP is a major regulator of caspases, we chose to further scrutinize the relationship between NRAGE and XIAP. NRAGE and XIAP have been shown to interact both by co-immunoprecipitation [26] and yeast two-hybrid [29] assays. However, we wanted to determine the endogenous distribution of intracellular expression of each protein in order to test whether they could be occupying similar subcellular locations, as would be expected if direct interactions are occurring. NRAGE was originally identified by our lab in the context of nerve cells [17]. Therefore, our experiments, when possible, were carried out in P19 cells, which are a known and accepted model for studying neuronal systems since they can be differentiated into neural- and glial-like cells in the presence of retinoic acid. We used an Alexa Fluor 488 IgG antibody to identify endogenous NRAGE (NRAGE-Alexa488) in fixed P19 cells and an Alexa Fluor 546 IgG antibody to identify endogenous XIAP (XIAP-Alexa546). Cells labeled with NRAGE-Alexa488 (Fig. 1A) and XIAP-Alexa546 (Fig. 1B) were imaged and the proteins were found to co-localize in the cytoplasm (Fig. 1C). Cells with neither Alexa488 nor Alexa546 were not fluorescent (data not shown).

### FRET shows direct interaction between endogenous NRAGE and XIAP

We have shown by immunoprecipitation experiments and Western blot analysis that NRAGE interacts with XIAP in P19 cells with and without BMP-4 treatment [26]. We sought to further test and refine our hypothesis, namely that NRAGE and XIAP are close enough to each other to bind directly, by using fluorescence resonance energy transfer (FRET), an established technique that relies on close (<10 nm) spatial proximity of fluorescent molecules (see Materials and Methods). With FRET, a “donor” fluorescent molecule can be quenched and cause fluorescence of an “acceptor” molecule if the respective proteins of interest tagged with each fluorescent molecule are close enough together such that the tags are within ∼10 nm of one another. Here, we assessed for FRET using donor dequenching [30], a method that determines the increase in donor fluorescence intensity (dequenching) caused by the loss of energy transfer when the acceptor photobleaches. The advantage of the donor dequenching method is that the parameters used to calculate the energy transfer efficiency can be determined from several images of the same field of cells, allowing each cell to be its own control, making this method straightforward and quantitative [31]. Evidence of increased donor fluorescence after acceptor photo bleachingphotobleaching indicates energy transfer between the donor and acceptor, which can only occur when the donor-acceptor distance is less than 10 nm, a distance that is likely too small to be occupied by another protein. Observation of FRET, therefore, would support our hypothesis of a direct interaction between NRAGE and XIAP.

**Figure 2:**
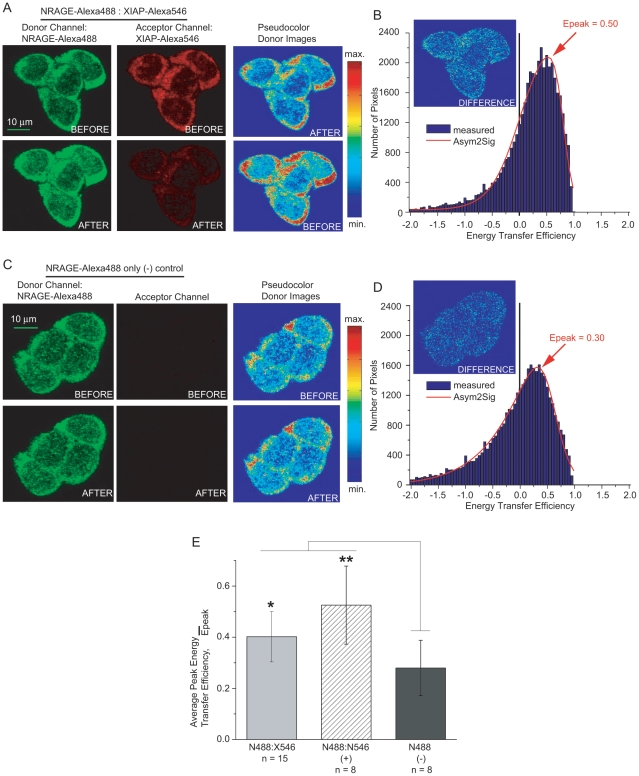
FRET analyses of the endogenous interaction between NRAGE and XIAP in P19 cells. (A) Confocal images of NRAGE-Alexa488 and XIAP-Alexa546 before and after acceptor photobleaching of Alexa Fluor 546 molecules showing an increase in fluorescence intensity of donor Alexa Fluor 488 molecules also evident by pseudocolor donor Alexa488 intensity images. (B) Asym2Sig fit (red line) of experimental histogram (blue bars) distribution energy transfer efficiencies of the donor images from (A) indicating a peak energy transfer efficiency of 50%. (C) Confocal and pseudocolor intensity images of donor only NRAGE-Alexa488 negative control showing little intensity change. (D) Corresponding histogram (blue bars) and fit (red line) of the negative control indicating a much lower energy transfer efficiency. (E) Average peak energy transfer efficiencies for endogenous NRAGE and XIAP interaction compared to positive and negative controls where *P<0.01 and **P<0.005; n is the number of images each containing 2 to 6 cells. N488, NRAGE-Alexa488; N546, NRAGE-Alexa546; X546, XIAP-Alexa-546.

Continuing with our approach in identifying endogenous NRAGE-Alexa488 and XIAP-Alexa546 in fixed P19 cells, we performed donor dequenching FRET analyses. Confocal images of NRAGE-Alexa488 were typically and noticeably brighter in fluorescence intensity after photo bleachingphotobleaching the Alexa546 used to detect XIAP (Fig. 2A, first and second columns) supporting the strong possibility of a direct interaction between NRAGE and XIAP. To quantify the intensity changes of Alexa488, pseudo-colorpseudocolor intensity donor images (Fig. 2A, third column) before and after acceptor photo bleachingphotobleaching were compared pixel-by-pixel, generating a histogram of intensity changes (Fig. 2B, blue bars) which was stored as a matrix. The matrix was imported into our data analysis software where it was fitted to characterize the statistical peak location (Fig. 2B, red line). The asymmetric double sigmoidal fitting function (Asym2Sig) was chosen because it was able to successfully fit all measured distributions. In this example, our analysis showed peak energy transfer efficiency, E_peak_, of 50±0.017% (Fig. 2C). Values for E at 50% indicate that the distance between donor and acceptor fluorophores (assuming an average over all dipole orientations) are equal to the Förster distance R_0_. Here, the calculated R_0_ is 6.0 nm for Alexa488:Alexa546 FRET (see Table 1), indicating that a direct interaction between endogenous NRAGE and XIAP is strongly suggested. We determined that FRET assessed by donor dequenching occurred at a significant level from over a dozen sets of confocal images containing 2 to 6 cells each. Average peak energy transfer efficiency, Ē_peak_, for endogenous NRAGE-Alexa488:XIAP-Alexa546 was 40±0.098%, significantly higher than our negative control (Fig. 2E). Theoretically, distances between donor and acceptor fluorophores of 2R_0_ lead to a drastically reduced energy transfer efficiency of 1.5%. Observation of a low energy transfer value (of a few percent or less) would be expected in cases where molecules do not interact and are spatially separated on the average. However, excitation can also be transferred from one fluorophore to another of the same kind (called homo-FRET) as seen experimentally in our negative control of endogenous NRAGE labeled with only donor Alexa488 and which we used as our baseline for lack of protein-protein interaction (Fig. 2C and D). The positive control was endogenous NRAGE doubly labeled with Alexa488 and Alexa546.

**Table 1:** FRET Distances, R_0_.

FRET Pair	Published R_0_	Calculated R_0_
Alexa488 : Alexa546	6.4 nm†	6.00 nm§
EGFP : DsRed	4.71±0.09 nm‡	5.05 nm§
ECFP : EYFP	4.92±0.10 nm¶	4.90 nm§

† Spence MTZ, ed. The Handbook A guide to Fluorescent Probes and Labeling Technologies: Invitrogen Corporation, 2005.

‡ Erickson MG, Moon DL, Yue DT. DsRed as a Potential FRET Partner with CFP and GFP. Biophysical Journal 2003; 85:599–611.

¶ Patterson GH, Piston DW, Barisas BG. Förster Distances between Green Fluorescent Protein Pairs. Analytical Biochemistry 2000; 284:438–40.

§ Discrepancies between published and calculated FRET distances are possibly due to the specific variability of the experimental environments, choice of published donor quantum yields and acceptor extinction coefficients, and source of fluorescent spectra intensities.

### EGFP:DsRed FRET indicates a direct interaction between the NRAGE WQxPxx repeat domain and XIAP

**Figure 3:**
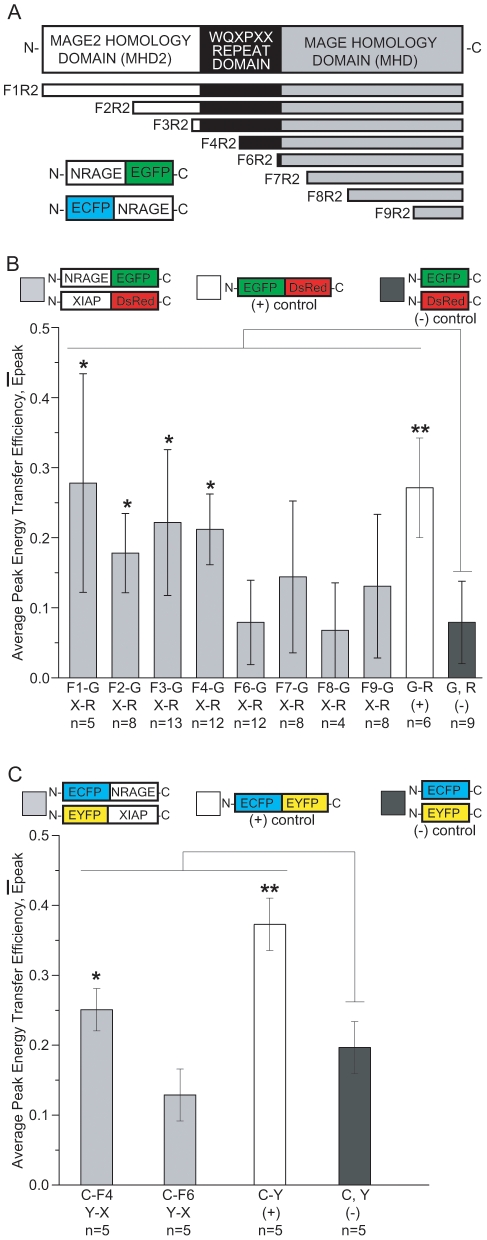
FRET efficiencies are higher between NRAGE constructs containing the repeat domain and XIAP than with NRAGE constructs without the repeat domain. (A) Schematic of NRAGE deletion mutations and their fusion scheme with EGFP and ECFP fluorescent tags. (B) Average peak energy transfer efficiencies between NRAGE-EGFP and XIAP-DsRed showing that full-length NRAGE (F1R2), F2R2, F3R2, and F4R2 have significantly higher FRET activity with XIAP than NRAGE F6R2, F7R2, F8R2, and R9R2 compared to the negative control. (C) Average peak energy transfer efficiencies of ECFP-NRAGE F4R2:EYFP-XIAP and ECFP-NRAGE F6R2:EYFP-XIAP recapitulating significant FRET and negligible FRET respectively with the EGFP:DsRed FRET in (B). *P<0.05, **P<0.005; n is the number of images each containing 1 to 3 cells. F1-G(NRAGE F1R2-EGFP), F2-G(NRAGE F2R2-EGFP), F3-G(NRAGE F3R2-EGFP), F4-G(NRAGE F4R2-EGFP), F6-G(NRAGE F6R2-EGFP), F7-G(NRAGE F7R2-EGFP), F8-G(NRAGE F8R2-EGFP), F9-G(NRAGE F9R2-EGFP); C-F4, NRAGE ECFP-F4R2; C-F6, NRAGE ECFP-F6R2; X-R, XIAP-DsRed; Y-X, EYFP-XIAP; G, EGFP; R, DsRed; C, ECFP; Y, EYFP.

NRAGE contains three domains – the MAGE2 homology domain (MHD2) at the N-terminus, the MAGE homology domain (MHD) at the C-terminus, and the repeat domain in the middle. Jordan et al identified three NRAGE clones that bind XIAP using a yeast two-hybrid screen, each of which contained the N-terminus through most of the repeat domain, or through the C-terminus, or in between [29]. We utilized our NRAGE-EGFP mutation series which delete from the N-terminus and keep the C-terminus intact [28] (Fig. 3A) and co-transfected each one with XIAP-DsRed into NIH3T3 cells, fixed, and analyzed for FRET. Examples of appreciable FRET (Fig. S1A) and negligible FRET (Fig. S1C) are shown for NRAGE F4R2-EGFP:XIAP-DsRed and NRAGE F6R2-EGFP:XIAP-DsRed respectively. Side-by-side evaluations of false-color and pseudo-colorpseudocolor images of NRAGE F4R2-EGFP:XIAP-DsRed show significant donor (EGFP) intensity enhancement after acceptor DsRed photo bleachingphotobleaching (Fig. S1A) while NRAGE F6R2-EGFP:XIAP-DsRed images show insignificant donor intensity enhancement (Fig. S1B). The fitted distribution of this example of NRAGE F4R2:XIAP had a peak energy transfer efficiency of 30±0.010% (Fig. S1B) compared to a peak energy transfer efficiency of just 3±0.018% for an example of NRAGE F6R2:XIAP (Fig. S1D).

Using our complete set of NRAGE-EGFP constructs, we found that the FRET results diverged into two groups – one showing appreciable FRET signals suggesting a direct interaction between NRAGE and XIAP and a second with low FRET signals suggesting apparent non-interaction. There were appreciable and statistically significant FRET signals between XIAP-DsRed and each of the NRAGE deletion mutations F1R2- (0.28±0.15%), F2R2- (0.18±0.05%), F3R2- (0.22±0.10%), and F4R2-EGFP (0.21±0.05%), similar to the EGFP-DsRed fusion positive control (0.27±0.07%) compared to the negative control (0.08±0.05%) in which EGFP and DsRed were co-transfected as separate plasmids with no fusion (Fig. 3B). NRAGE F1R2, F2R2, F3R2, and F4R2 each retain at least half of the repeat domain through to the end of the C-terminus whereas the remaining group of NRAGE-EGFP constructs eliminates most of the repeat domain. Interestingly, this group also showed a significant percentage of early apoptosis by Annexin V positive staining in our previous work [28]. FRET signals were not significant between XIAP-DsRed and each NRAGE F6R2- (0.08±0.06%), F7R2- (0.14±0.11%), F8R2- (0.07±0.06%), and F9R2-EGFP (0.13±0.10%) in which there is no repeat domain or only very little (6 amino acids in F6R2) similar to the negative control (Fig. 3B). This group also had a significantly lower incidence of Annexin V staining [28]. Overall, there was some cell-to-cell variability in FRET signals with some cells showing significant positive FRET signals and some showing low FRET signals. This may be explained by the binding of other members of the NRAGE-XIAP complex, namely TAB1 and TAK1, that could be responsible for bringing NRAGE and XIAP in inconsistent proximity to each other, with the low FRET signals meaning that there is likely to be too great a distance for a direct interaction.

### ECFP:EYFP FRET recapitulates that the NRAGE repeat domain interacts with XIAP

We concentrated our efforts on the repeat domain of NRAGE, specifically the F4R2 and F6R2 partition, as a critical point in the NRAGE-XIAP interaction because of the significantly higher FRET signal of XIAP for F4R2 than F6R2 and to provide additional evidence on the importance of the NRAGE repeat domain. To recapitulate that NRAGE F4R2 and F6R2 is where interaction with XIAP takes place and does not take place respectively, we decided to again use donor dequenching FRET, but this time by the well known FRET pair CFP and YFP. Additionally, we wanted to know whether fusing the fluorescence tags to the N-termini of NRAGE and XIAP would adversely affect their binding as compared to the EGFP and DsRed constructs which were fused to the C-termini. 293T cells were co-transfected with either ECFP-NRAGE F4R2 and EYFP-XIAP or ECFP-NRAGE F6R2 and EYFP-XIAP before being fixed and imaged for FRET (see Fig. S2 for examples). Typically, cells showed a higher and significant average FRET efficiency signal between ECFP-NRAGE F4R2 and EYFP-XIAP (0.25±0.03%) than for ECFP-NRAGE F6R2 and EYFP-XIAP (0.12±0.04%) when compared to the CFP-YFP fusion positive control (0.37±0.04%) and CFP:YFP negative control (0.19±0.04%) (Fig. 3C). Consistent with the outcome of the EGFP:DsRed FRET, this shows that the NRAGE repeat domain is necessary for XIAP interaction.

### The NRAGE repeat domain is required for NF-κB activation in the non-canonical BMP pathway

**Figure 4:**
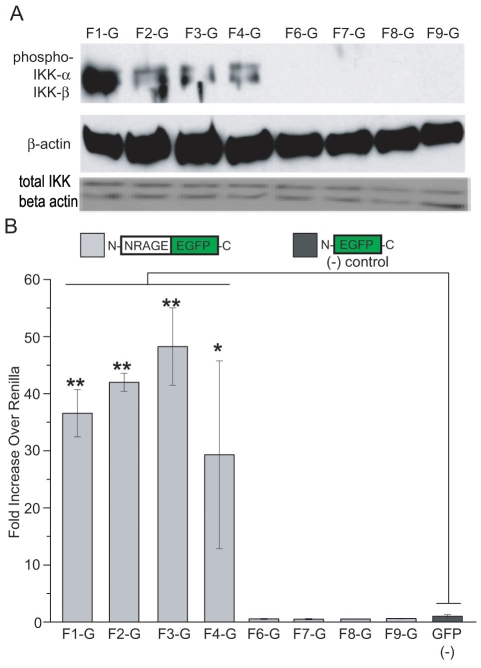
Activation of the NF-κB pathway requires the NRAGE repeat domain in the BMP pathway. (A) HEK293 cells were transfected with the NRAGE-EGFP constructs and western blotted for IKK-α/β-phosphorylation and total IKK. (B) NRAGE-EGFP transfected HEK293 cells were also assessed for NF-κB activation by Luciferase assay. *P<0.05, **P<0.005. F1-G(NRAGE F1R2-EGFP), F2-G(NRAGE F2R2-EGFP), F3-G(NRAGE F3R2-EGFP), F4-G(NRAGE F4R2-EGFP), F6-G(NRAGE F6R2-EGFP), F7-G(NRAGE F7R2-EGFP), F8-G(NRAGE F8R2-EGFP), F9-G(NRAGE F9R2-EGFP).

In light of our FRET results strongly suggesting that the repeat domain was the key domain for XIAP interaction, we wanted to determine if this domain was also critical for downstream signaling. Because the BMP pathway is also known to regulate the nuclear factor NF-κB through XIAP-TAB1-TAK1 [32], [33], [34], we questioned what effect the NRAGE deletion mutations might have on this transcriptional activation. In unstimulated cells, NF-κB is sequestered in the cytoplasm by κB inhibitors. However, when activated by signals often from outside of the cell, IκB kinase (IKK) becomes activated which induces degradation of κB inhibitors thus freeing NF-κB for nuclear translocation and transcription of target genes. Constitutive phosphorylation of IKK-α and IKK-β subunits was found in only the WQxPxx repeat domain-containing constructs F1R2 through F4R2 (Fig. 4A). In addition, we evaluated the transcriptional activity of NF-κB by NF-κB-luciferase reporter vector after transfection of the NRAGE-EGFP constructs in HEK293 cells. Similar to the western blot data (Fig. 4A), transfection with full length NRAGE F1R2 and repeat domain-containing constructs F2R2 through F4R2 resulted in constitutive NF-κB transcriptional activation (Fig. 4B), resulting in a 10 to 60 fold induction compared to the GFP control. NRAGE mutant constructs which did not contain the MAGE2 homology domain or the repeat domain (F6R2 through F9R2) did not activate the NF-κB pathway.

### Isolated NRAGE repeat domain FRETs with XIAP

**Figure 5:**
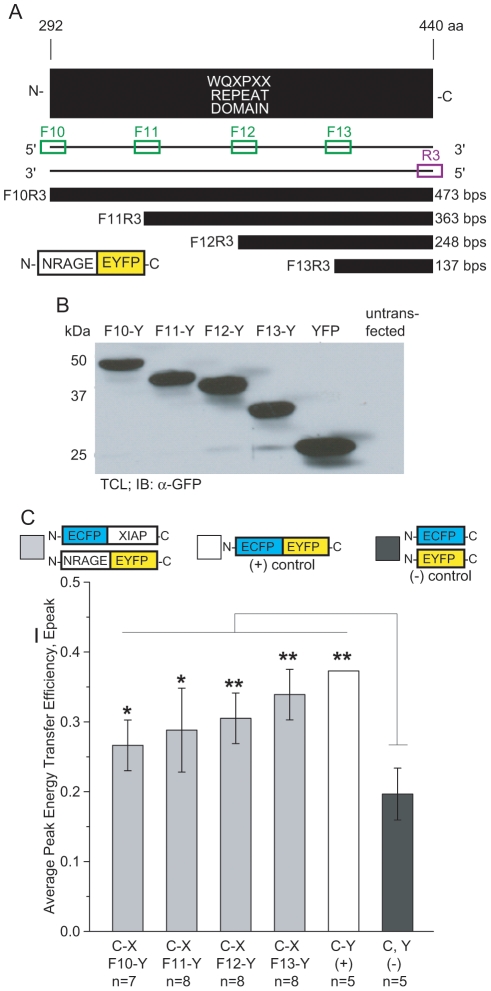
NRAGE repeat domain deletion mutations and XIAP interaction by FRET. (A) Schematic of 4 designs of the repeat domain in NRAGE denoted by their primers as F10R3, F11R3, F12R3, and F13R3 and their fusion scheme with a EYFP fluorescent tag and (B) protein expression in P19 total cell lyses. (C) Peak energy transfer efficiency averages show progressively increasing FRET signals between ECFP-XIAP and NRAGE repeat domain deletions-EYFP when compared to controls (*P<0.05; **P<0.005; n is the number of images each containing 1 to 3 cells). C-X, ECFP-XIAP; Fx-Y, NRAGE FxR3-EYFP.

We proceeded to investigate only the repeat domain of NRAGE and its interaction with XIAP because the NRAGE constructs containing at least half the repeat domain (F1R2 through F4R2) yielded reproducible FRET results. We created deletion mutations of only the NRAGE repeat domain, tagging each one with EYFP at the C-terminus of NRAGE (Fig. 5A and B). To evaluate direct interaction with XIAP, we co-transfected each YFP-tagged NRAGE mutant repeat domain along with CFP-XIAP into 293T cells and performed donor-dequenching FRET imaging and analyses. Significant FRET was observed between each NRAGE repeat domain-EYFP and ECFP-XIAP. In fact, the FRET efficiency increased progressively with each sequentially smaller NRAGE repeat domain (Fig. 5C) meaning that the distance between ECFP and EYFP fluorescent tags was decreasing due to the apparent direct binding between decreasing NRAGE repeat domain deletions and XIAP. Peak average energy transfer efficiencies were measured at 0.27±0.04% for ECFP-XIAP:F10R3-EYFP, 0.29±0.06% for ECFP-XIAP:F11R3-EYFP, 0.31±0.04% for ECFP-XIAP:F12R3-EYFP, and 0.34±0.04% for ECFP-XIAP:F13R3-EYFP.

### A small peptide modeled after the NRAGE repeat domain inhibits interactions between BMP MAPK members

**Figure 6:**
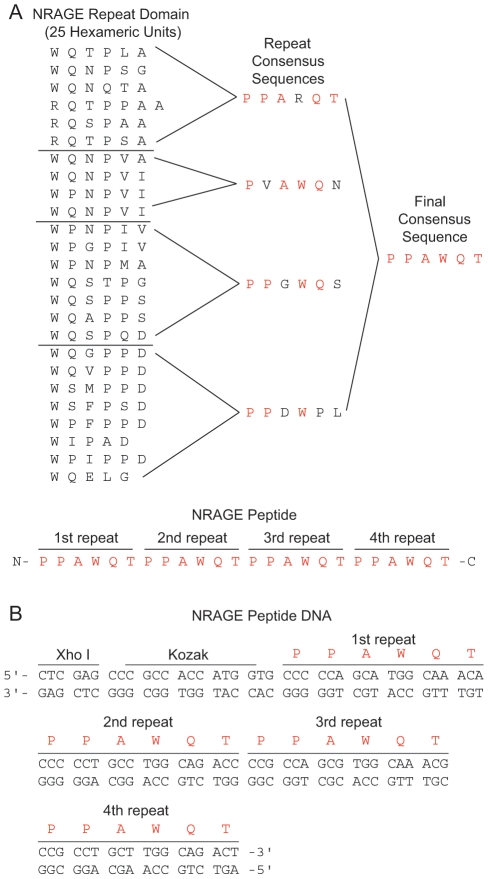
Design of the NRAGE peptide. (A) A list of the 25 hexamers in the order in which they appear in the NRAGE repeat domain partitioned into 4 distinguishing groups. A repeat consensus sequence was determined for each group and was then resolved into a single final consensus sequence as indicated by the red amino acids which was repeated 4 times for the NRAGE peptide. (B) Schematic of nucleotides comprising the NRAGE peptide including a Xho I site and a Kozak sequence to facilitate excision and transcription respectively.

**Figure 7:**
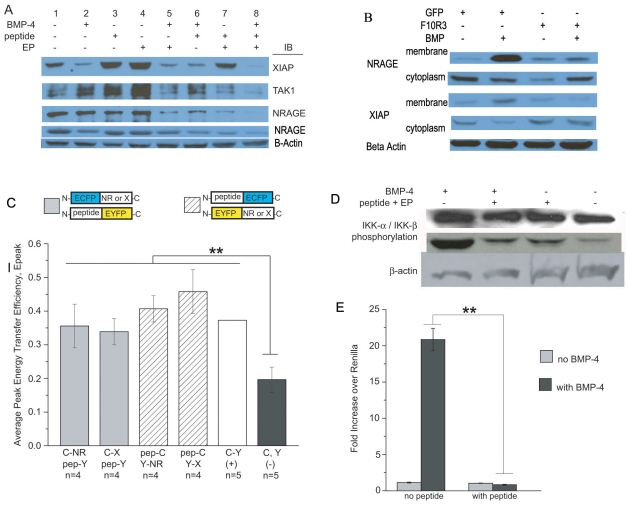
The NRAGE peptide inhibits some cytoplasmic BMP MAPK pathway members, binds to NRAGE and XIAP, and inhibits downstream activation of NF-κB and IKK. (A) Western blot of endogenous expression of NRAGE, XIAP, and TAK1 in P19 cells after XIAP or NRAGE immunoprecipitation in lyses and NRAGE and Beta-Actin expression of total cell lysates from cells that underwent BMP-4 treatment with and without the NRAGE peptide compared to untreated cells. (B) CFP:YFP FRET assessment of CFP or YFP tagged NRAGE peptide, full-length NRAGE, and XIAP in fixed 293T cells. (C) Western blotting shows that activation of IKK by BMP-4 in HEK293 cells is inhibited in cells cultured with the NRAGE peptide. (D) NF-κB activation is inhibited in HEK293 cells cultured with the NRAGE peptide compared to HEK293 cells that did not receive the peptide as assessed by luciferase assay. Treatments were 10 ng/ml BMP-4, 10 nM NRAGE peptide, and 6 µM EndoPorter delivery reagent where indicated. **P<0.005. C-NR, CFP-NRAGE; Y-NR, YFP-NRAGE; C-X, CFP-XIAP; Y-X, YFP-XIAP; pep-C, NRAGE peptide-CFP; pep-Y, NRAGE peptide-YFP. (E) GFP control P19 cells and the F10R3 NRAGE repeat domain P19 cells treated with and without 10 ng/ml BMP-4, then cell membranes and cytoplasm ran seperatly to see the affects of XIAP and NRAGE expression. Beta Actin was used as loading control.

We wanted to further exploit the NRAGE repeat domain and designed a small peptide mimetic modeled after this domain (see Materials and Methods and Fig. 6). As shown in Figure 7A, we used western blotting of cytoplasmic endogenous NRAGE immunoprecipitates from P19 cells to evaluate the interactions of non-canonical BMP members. XIAP was much more easily detected in cells that did not receive BMP-4 (Fig. 7A, lanes 1, 3, 4, and 7) than cells that did receive BMP-4 (Fig. 7A, lanes 2, 5, 6, and 8). However, TAK1 was hardly detectable in BMP-4 treated cells that received a simultaneous treatment of NRAGE peptide and EndoPorter delivery reagent (Fig. 7A, lane 8) than in any other treated cells (Fig. 7A, lane 1 through 7). When cells were exposed to BMP-4, detection for XIAP and TAK1 diminished in peptide/EndoPorter treated cells (Fig. 7A, lane 8) compared to cells without the NRAGE peptide (Fig. 7A, lane 2). NRAGE was easily detected in cells that were untreated, or received BMP-4, or the NRAGE peptide alone, or EndoPorter alone(Fig. 7A, lanes 1 through 4), but was greatly diminished in cells treated with BMP-4 and EndoPorter, BMP-4 and NRAGE peptide, and NRAGE peptide/EndoPorter (Fig. 7A, lanes 5 through 7); and similar to XIAP and TAK1, hardly detectable in cells that received BMP-4 and NRAGE peptide/EndoPorter (Fig. 7A, lane 8). This suggests that complexes that are comprised of NRAGE, XIAP, and TAK1 do not reside in the cytoplasmic compartment.

Efforts were made to see if culturing with the peptide could inhibit the interaction of overexpressed fluorescently tagged full-length NRAGE and XIAP using donor dequenching CFP:YFP FRET, but no inhibition was evident at peptide concentrations of 10 nM, 100 nM, nor 1 µM (data not shown). It is possible that introducing a fixed amount of the NRAGE peptide to the cells with EndoPorter is not enough to compete with the co-transfection and resulting constituitive expression of NRAGE and XIAP. Therefore, we created the DNA equivalent of the NRAGE peptide and tagged it with either CFP or YFP (see Materials and Methods and Fig. 6B) for delivery into cells by transfection to see if it was binding directly to full-length NRAGE or XIAP by CFP:YFP FRET assessment. Co-transfections of the NRAGE peptide DNA with NRAGE showed very favorable energy transfer efficiencies of 0.36±0.06% and 0.41±0.04% while co-transfections of the NRAGE peptide DNA with XIAP also showed significant energy transfer efficiencies of 0.34±0.04% and 0.46±0.06% when compared to the negative control (Fig. 7B) meaning that the NRAGE peptide binds directly to both NRAGE and XIAP. It is possible that binding could be occurring in either NRAGE peptide-XIAP, NRAGE peptide-NRAGE, or NRAGE peptide-NRAGE-XIAP sub-complexes and that there may be a certain number of sub-complexes needed to initiate downstream signaling, since it has been shown that Xiap diamerizes in the XIAP-TAK-TAB complex and that disruption of the diamerization leads to the inhibition of downstream signaling [33].

### The NRAGE peptide inhibits downstream activation of NF-κB

We showed that IKK-α/β is phosphorylated in HEK293 cells transfected with NRAGE-EGFP constructs F1R2 through F4R2 which contain the repeat domain (Fig. 4). We wanted to determine if culturing with the NRAGE peptide could deter IKK phosphorylation. Western blotting of lysates from HEK293 cells that received the NRAGE peptide in the presence of BMP-4 show minimal detection of phosphorylated IKK (Fig. 7C, lane 2) when compared to cells stimulated by BMP-4 without the peptide (Fig. 7C, lane 1). Detection of phospho-IKK-α/β was negligible in cells not stimulated by BMP-4 whether or not they received the peptide treatment (Fig. 7C, lanes 3 and 4) confirming that stimulation by BMP or NRAGE overexpression is needed to activate IKK. In addition, NF-κB activation was reduced from 20.85±1.55 fold increase over renilla in HEK293 cells cultured without the NRAGE peptide to 0.85±0.06 when cultured with the NRAGE peptide and after being stimulated with BMP-4 (Fig. 7D).

### The NRAGE peptide DNA inhibits apoptosis in P19 cells

**Figure 8:**
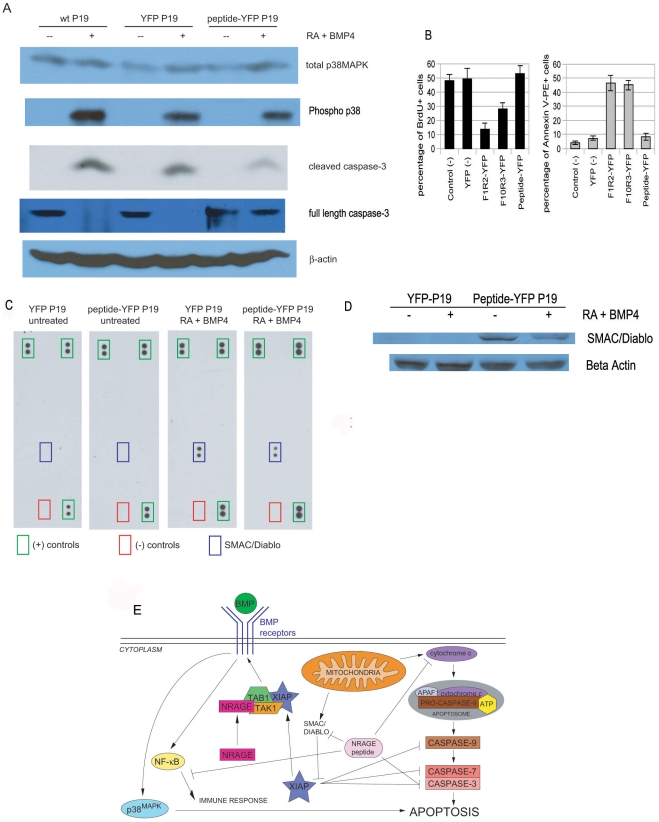
Apoptosis is inhibited in P19 cells cultured with the NRAGE peptide. (A) Western blot of P19 cells transfected with the NRAGE peptide-YFP had less detection of cleaved and more full-length caspase-3 compared to wild-type or YFP transfected cells after 24 hours exposure of 1 µm RA and 10 ng/ml BMP-4. c. Higher levels of total-p38MAPK are detected in all cells that received apoptosis treatment. β-actin was used for loading control. (B) Stably integrated YFP and NRAGE peptide-YFP cells without retinoic acid (RA) and BMP-4 were lysed and incubated to human apoptosis profiler antibody nitrocellulose arrays (left 2 panels). Arrays were stripped and semi-stable YFP and NRAGE peptide-YFP cells incubated with 1 µM RA+10 ng/ml BMP-4 for 24 hours were lysed and incubated to the same respective arrays and found to bind with SMAC/Diablo, but with less detection for NRAGE peptide-YFP cells. (C) Western blot analysis of total cell lysate of SMAC/Diablo expression of YFP and NRAGE peptide-YFP cells with and without BMP-4 stimulation. (D) Annexin V and BRDU flow analysis of YFP control cells, NRAGE full length, NRAGE repeat, and NRAGE-peptide cell lines (E) Proposed signaling BMP MAPK pathway incorporating the inhibition of SMAC/Diablo by the NRAGE peptide resulting in the inhibition of cleaved caspase-3 and apoptosis.

Retinoic acid (RA) and BMP-4 each alone induce apoptosis in P19 neural progenitor cells and in combination induce death in up to 40% of the cell population in as little as 24 hours of exposure [35], [36]. Overexpression of NRAGE has a similar effect to RA and BMP-4 co-treatment on apoptosis in P19 cells [37]. Survival following RA and BMP-4 co-treatment can be increased to untreated levels by suppressing NRAGE expression [26]. Therefore, we sought to determine if the NRAGE peptide could inhibit RA and BMP-4 induced apoptosis in P19 cells by measuring levels of cleaved caspase-3, a protein of the caspase family that plays a central role in the execution phase of cell apoptosis. Correlating with our previous work that apoptosis in P19 cells occurs through p38^MAPK^ signaling, total p38^MAPK^ was more evident in cells that received RA and BMP-4 treatment then cells without treatment (Fig. 8A). We transfected fluorescently tagged NRAGE peptide-YFP DNA and YFP control DNA into P19 cells then induced apoptosis with RA and BMP-4, collected total cell lysates, and immunoblotted for cleaved and full-length caspase-3. As shown in Figure 8A, less cleaved caspase-3 and higher levels of full-length caspase-3 could be detected in NRAGE peptide-YFP transfected P19 cells than YFP control transfected or untransfected wild-type P19 cells. We also show a significant decrease in phospho-p38 expression of the BMP-4 induced peptide-YFP cells.

We wished to determine if there were any other pro- or anti-apoptotic proteins that may be expressed or inhibited in P19 cells pre-treated with the NRAGE peptide. To this end, we used cell lysates from established stably-integrated NRAGE peptide-YFP and YFP control P19 cell lines on human apoptosis protein antibody profiler antibody arrays to detect pro- and anti-apoptosis proteins with and without RA and BMP-4. Except for positive controls, no proteins were identified in the absence of apoptosis induction in YFP control cells nor NRAGE peptide-YFP cells (Fig. 8B, left two arrays). However, when both cell lines were exposed to RA and BMP-4 for 24 hours, SMAC/Diablo was detected in both with lower levels in NRAGE peptide-YFP cells (Fig. 8B, right two arrays and Fig. 8C). SMAC/Diablo is a mitochondrial protein that is released during apoptosis and promotes cytochrome c-dependent caspase activation by neutralizing IAPs [39], [40]. The decreased detection of SMAC/Diablo in NRAGE peptide-YFP cells suggests that the NRAGE peptide may be preventing apoptosis by preserving the integrity of the mitochondrial outer membrane (Fig. 8C). To give further evidence that the nrage peptide alters the apoptotic affects, we show that compared to full length NRAGE-YFP or the NRAGE repeat domain the NRAGE peptide-YFP cell line shows a decreased affect on apoptosis and an increase in proliferation Ifig 8D).

## Discussion

Apoptosis is a form of programmed cell death which takes place in all organisms during normal development and regular maintenance, preserving tissue homeostasis. However, in situations where it functions uncontrollably, it can endanger survival of the organism by either causing too much cell death as in neurodegenerative disorders or to too little cell death as in some cancers.

We initially identified NRAGE as a binding partner with p75^NTR^ that not only blocks binding between p75^NTR^ and TrkA to override apoptosis, but also facilitates cell cycle arrest and nerve growth factor-dependent apoptosis in sympathetic neuron precursor cells [17]. However, NRAGE is also expressed throughout embryonic and adult tissues in a spatial and temporal pattern independent of p75^NTR^ expression, suggesting an alternative role [22]. NRAGE is a member of the MAGE family containing two MAGE homology domains and a third repeat domain made up of 25 hexameric peptides with a consensus sequence of WQxPxx that is only consistent with NRAGE homologs in mouse, rat, and human. Using our NRAGE-EGFP deletion mutations, we found that apoptosis in P19 cells was significantly reduced without the repeat domain [28]. BMPs play profound roles in regulation of neural differentiation, apoptosis, and dorsal-ventral patterning. NRAGE interacts with BMP MAPK members XIAP, TAK1, and TAB1, which form a complex that interacts with BMP receptors facilitating TAK1 transduction signals [41]. The NRAGE-XIAP-TAB1-TAK1 complex enables BMP-mediated apoptosis in cortical neural progenitors, neural differentiated P19 cells, and undifferentiated P19 cells, which can be correlated to increases in the well known apoptotic indicators cleaved caspase-3 and phosphorylated p38^MAPK^ [26], [28]. XIAP can inhibit apoptosis by binding with caspases and interrupting their activation through their BIR domains [13], [14]. Therefore, we focused our efforts on the binding between NRAGE and XIAP.

Previously, FRET has been used in XIAP signaling to quantify the distance between the BIR2 and BIR3 domains in determining the additional hydrophobic binding surface area versus the BIR3 domain alone for the enhancement of a SMAC peptide mimetic that *enhances* caspase-driven apoptosis for cancer therapeutics [42]. We report here the first time FRET has been used to not only show a high probability of a direct interaction between NRAGE and XIAP, but also to map the interacting site to the NRAGE repeat domain. We utilized our inventory of NRAGE-EGFP deletion mutations to determine that the NRAGE repeat domain is critical for XIAP binding and developed an NRAGE peptide mimetic that *inhibits* caspase-driven apoptosis. XIAP is also known to direct the activity of NF-κB as it is an essential component in TGF-β signaling that stimulates NF-κB in metastatic 4T1 breast cancer cells [43]. Conversely, XIAP- and TAK1-TAB1-mediated NF-κB activation can be inhibited by Siva1 to enhance apoptosis by JNK activity [34]. We also determined that the NRAGE repeat domain is responsible for NF-κB activation which can be inhibited by the NRAGE peptide. NRAGE overexpression induces activation of caspases-3, -9, and -7, and caspase dependent cell death in a JNK-dependent mitochondrial pathway to facilitate p75^NTR^-mediated cell death in PC12 cells [38]. Our data shows that culturing with the NRAGE peptide before inducing apoptosis can alleviate caspase-3 activation and reduce p-38 phosphorylation. This may be applicable in attenuating p75NTR-dependent death which occurs in oligodendrocytes and hippocampal neurons [44], [45].

The endogenous prion protein (PrP) is predominantly an extracellular glycosyl-phosphatidyl-inositol-anchored protein whose function is unknown, but whose conversion to a disease-associated form leads to prion diseases such as Creutzfeldt-Jakob disease in humans, Scrapie in sheep, and Bovine Spongiform Encephalopathy in cattle. A subset of PrP is present in the cytosol and may have a physiological function in apoptosis regulation depending on the neuronal cell type and its context. PrP binds to the repeat region of NRAGE and affects mitochondrial membrane potential which can lead to apoptosis [19]. Therefore, application of the NRAGE in this realm may provide important information in the investigation of preventing prion diseases.

In the future, we intend to resolve how the NRAGE peptide is functioning to inhibit BMP MAPK pathway activation. For example, if it is binding to either NRAGE or XIAP; or if it is binding to both, but in separate complexes. Lu et al. have determined that XIAP-TAB1-TAK1 form a dimer that transduces downstream signaling for NF-κB. However, mutation of XIAP's BIR1 domain disrupts dimerization resulting in downstream inhibition NF-κB signaling [33]. Therefore, it is also possible that culturing with the NRAGE peptide is also disrupting XIAP-TAB1-TAK1 dimerization resulting in the downstream inhibition of NF-κB activation and yielding protective affects for apoptosis-induced cells while possibly allowing some binding to occur between NRAGE, XIAP, TAB1, and TAK1.

## Materials and Methods

### NRAGE cloning

NRAGE-EGFP deletion mutations F1R2 to F9R2 were made as previously described [28] and are denoted as NRAGE FxR2-EGFP where ‘x’ is ‘1’ through ‘9’ for the respective deletion mutation. NRAGE F5R2 was not accomplished. NRAGE F4R2 and F6R2 were also PCR'd from EGFP-N3 constructs [28] using HotMasterMix (Eppendorf, Hamburg, Germany) and cloned into pGEM-T Easy vector (Promega, Madison, WI) and then into pECFP-C1 (Clontech/Takara Bio, Mountain View, CA) using EcoR I and Sal I restriction enzymes so that the C-terminus of ECFP was fused to the N-terminus of NRAGE (denoted as ECFP-FxR2 NRAGE where “x” is the appropriate deletion mutation).

NRAGE F10R3 to F13R3 repeat domain fragments were PCR'd from full-length NRAGE-EGFP made previously [28] using HotMasterMix (Eppendorf) according to Fig. 5A and linearly decrease by 1/4 the repeat domain from F10R3 to F11R3 to F12R3 and to F13R3 with F10R3 being the full-length repeat domain. Forward primers contain a Kozak consensus sequence to facilitate translation [46], [47] and were 5′-CCCGCCACCAT GGGGCAGACACCACTGGCT-3′ for F10, 5′-ACCGCCACCATGGGGCAGAACCCA GTTGCA-3′ for F11, 5′-ATCGCCACCATGGACCCAATGGCCTGGCAG-3′ for F12, and 5′-GACGCCACCATGGCACCTGACTGGTCAATG-3′ for F13. Reverse primer R3 was 5′-CAGATTAGTCGACGGTCGTAAGTTCT GCCA -3′. All PCRs were done at 1 minute at 94°C incubation; 30 cycles of 30 seconds at 94°C denaturing, 30 seconds at 58°C annealing, and 30 seconds at 65°C extension; and a final incubation of 4°C. Each PCR product was cloned into pGEM-T Easy vector (Promega) and then into pEYFP-N1 (Clontech) using EcoR I and Sal I restriction enzyme sites so that the N-terminus of EYFP was fused to the C-terminus of NRAGE (denoted as NRAGE FxR3-EYFP where “x” is the appropriate deletion mutation).

All ECFP, and EYFP constructs were verified by sequencing and by exhibiting fluorescence emitted at the respective wavelengths as viewed from an Axiovert 200 microscope (Carl Zeiss, Göttingen, Germany). Fluorescent filters were 436/20 nm excitation, 455 nm dichroic, 480/40 nm emission for ECFP (Chroma, Bellows Falls, VT) and 500/20 nm exciter, 515 nm dichroic, 535/30 nm emission for ECFP (Chroma).

### XIAP cloning

XIAP accession # U88990 (National Center for Biotechnology Information) was PCR'd using forward primer 5′-GTCCTATTTTCAAGAAT TCATGACTTTTAACAG-3′, reverse primer 5′-TGCCTACTATAGAGTCCCGGGAAGACATAAAAA-3′, and HotMasterMix (Eppendorf). PCR cycles were 1 min., 94°C incubation; 30 sec., 94°C denature; 30 sec., 51°C annealing; and 1 min. 30 sec., 65°C extension for 30 cycles. XIAP PCR product was cloned into either pCR-Blunt II-TOPO vector (Invitrogen, Carlsbad, CA) or pGEM-T Easy vector (Promega) and then cloned into pDsRed-Monomer, pECFP, or pEYFP (Clontech) such that XIAP-DsRed has the fluorescent tag downstream to XIAP while ECFP- and EYFP-XIAP have the fluorescent tag upstream. All constructs are of full-length XIAP and were verified by immunoblotting with rabbit anti-XIAP antibody (Cell Signaling) and the Axiovert 200 microscope (Zeiss) for fluorescence using the fluorescent filters above and 565/30 nm excitation, 585 nm dichroic, 620/60 nm emission for DsRed (Zeiss).

### Cell cultures

NIH3T3, HEK293, 293T, and P19 cells were purchased from American Type Culture Collection (ATCC), Manassas, VA, and maintained according to ATCC specifications. For P19 cells stably expressing NRAGE peptide-YFP or control YFP vector, P19 cells were transiently transfected with NRAGE peptide-YFP or YFP control vector DNA using Lipofectamine 2000 (Invitrogen) following manufacturer's protocol. YFP+ cells were selected by culturing with P19 complete medium containing G418 antibiotic (Sigma).

### Immunocytochemistry for fluorescence and confocal microscopy for FRET

P19 cells were seeded at 40,000 cells per well in an 8-well chamber #1.5 German borosilicate coverglass systems (8-well Nunc; Nalge Nunc, Langenselbold, Germany) pre-treated with 0.01% poly-D-lsine. 24 hours later cells were rinsed 3× with DPBS, fixed and permeabilized with −20°C methanol for 10 minutes, and rinsed again 3× with DPBS. Cells were blocked with 0.1% Nonidet P-40 and 1% goat serum in DPBS and incubated at room temperature for one hour. Rabbit antiserum anti-NRAGE antibody (Upstate Biotechnolgy) and mouse monoclonal anti-XIAP antibody (R & D Systems) were applied at 1∶200 each in blocking solution and incubated at 4°C overnight. Cells were rinsed 3× with DPBS and secondary goat anti-rabbit Alexa Fluor 488 IgG and goat anti-mouse Alexa Fluor 546 IgG (Invitrogen) were applied at 1∶1000 and incubated for four hours in darkness at room temperature followed by rinsing 3× with DPBS. For FRET, cells were left hydrated in DPBS and imaged for FRET. Images of co-localization were obtained using the inverted Axiovert 200 microscope (Zeiss), a 63×/0.75NA air objective, and a CoolSnap HQ camera (Photometrics, Tucson, AZ). Fluorescent filters were 470/40 nm excitation, 495 nm dichoric, 525/50 nm emission for Alexa Fluor 488 (Zeiss). The DsRed filter cube used to verify XIAP-DsRed construct was also used for Alexa Fluor 546.

### Transfections for FRET

NIH3T3 cells were seeded in 96-well tissue CellStar culture treated plates (USA Scientific, Ocala, FL) at 20,000 cells per well in antibiotic free OPTI-MEM medium (Invitrogen) and co-transfected 24 hours later with 200 ng NRAGE-EGFP and 400 ng XIAP-DsRed using Lipofectamine 2000 (Invitrogen) for 4–5 hours before replacing the transfection medium with complete culture medium. Twenty-four hours post-transfection, cells were trypsinized and transferred to 8-well chamber #1.5 German borosilicate coverglass systems (8-well Nunc; Nalge Nunc, Langenselbold, Germany). After 48 hours post-transfection (to allow for full maturation of fluorophores), cells were rinsed 3× with DPBS, fixed with 4% paraformaldehyde for 20 minutes at room temperature, rinsed 3× with DPBS, and left hydrated in DPBS.

293T cells were seeded in 96-well tissue CellStar culture treated plates (USA Scientific) at 20,000 cells per well in antibiotic free culture medium and co-transfected 24 hours later using Lipofectamine 2000 (Invitrogen) left on overnight. For NRAGE F4,6R2 and XIAP experiments, cells were co-transfected with 200 ng ECFP-NRAGE and 400 ng EYFP-XIAP. For NRAGE F10,11,12,13R3 and XIAP experiments, cells were co-transfected with 400 ng ECFP-XIAP and 200 ng NRAGE-EYFP. 24 hours post-transfection, cells were trypsinized and transferred to an 8-well Nunc pre-treated with 0.01% poly-D-lysine. 48 hours post-transfection, cells were rinsed 3× with DPBS, fixed with 4% paraformaldehyde for 20 minutes at room temperature, rinsed 3× with DPBS, and left hydrated in DPBS.

### Luciferase assay

HEK293 cells plated at a density of 30,000 cells/well in 24 well culture plates were transfected with NF-κB-firefly luciferase and *Renilla* luciferase control plasmid (Stratagene) via GeneJuice (EMD Biosciences). Cells were then incubated with 3 µl EndoPorter and varying concentrations of NRAGE peptide for 48 hours. Cells were then serum starved for 4 hours prior to stimulation with 10 ng/ml BMP-4 for 24 hours. The Dual Luciferase Assay Kit (Promega) was used for the analysis of NF-κB transcriptional activity. All data are presented as a fold increase of NF-κB over *Renilla* activity and were performed in triplicate.

### Confocal imaging

For EGFP/DsRed NIH3T3 cells and Alexa Fluor P19 cells, cells were imaged using a TCS NT confocal system (Leica, Bannockburn, IL). Excitation for EGFP was at 488 nm from an Argon+ laser and for DsRed at 568 nm from a Krypton laser and collected from a 63×/1.2NA water immersion objective using a 488/568 nm double dichroic and either a 580 nm reflection short pass filter and 525/50 nm band pass filter for EGFP or Alexa Fluor 488, or a 650 nm reflection short pass filter and 590 nm long pass filter for DsRed or Alexa Fluor 546.

For ECFP/EYFP 293T cells, cells were imaged using a TCS SP confocal system (Leica). Excitation for ECFP was from a 440 nm diode laser and for EYFP an Argon+ laser selected for 514 nm and collected from a 63×/1.32NA oil immersion objective using acousto-optic tunable filters set for 465–495 nm emission for ECFP and 535–565 nm emission for EYFP.

### FRET experiments

Minimal laser power was used for donor (EGFP, Alexa Fluor 488, or ECFP) excitation to minimize photodamage in the donor channel and decrease spectral bleedthrough into the acceptor channel (DsRed, Alexa Fluor 546, or EYFP). Detector sensitivity for the acceptor channel was reduced to the point where visual detection was negligible using donor excitation. Donor dequenching was used to measure energy transfer from the increase in donor fluorescence after photobleaching of the acceptor fluorophore. Photobleaching of acceptors DsRed and EYFP required approximately 2–3 minutes and Alexa546 required approximately 10 minutes. Single-acquisition confocal images of cells using either donor or acceptor excitation before and after acceptor photobleaching were captured as ‘.tif’ documents and imported into Matrix Laboratory (MATLAB) software release R2007b (Mathworks, Natick, MA). Backgrounds of donor and acceptor images were determined by Adobe PhotoShop 7.0 software (Adobe Systems, San Jose, CA). Donor images before and after acceptor photobleaching were translated slightly along the X and Y axes for alignment if necessary before comparing background-subtracted fluorescence on a pixel-by-pixel basis. If there was focal plane drift (Z axis) between the single-acquisition donor pre- and post-bleaching images, then alignment was compensated for by either choosing the donor post-bleaching image acquired from a Z-stack that was most similar to the single-acquisition pre-bleach donor image or choosing the donor pre-bleaching image acquired from a Z-stack that was most similar to the single-acquisition post-bleach donor image. After compensation for background in the donor and acceptor channels, experimental energy transfer efficiency was calculated as E_exp_ = (F_D_−F_DA_)/F_D_ where F_D_ is the donor fluorescence without the acceptor and F_DA_ is the donor fluorescence with the acceptor. Intensity images were generated by MATLAB pseudocolor mapping and intensity differences in pixels were binned in 0.05 increments, plotted as a histogram, and stored as a matrix. The matrix was imported into Microcal Origin software (OriginLab, Northampton, MA) and fitted to the following asymmetric double sigmoidal (Asym2Sig) distribution
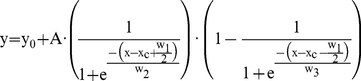
(1)where y_0_ is the offset of the fit from the y-axis; x_c_ is the center of the fit on the x-axis; A is the amplitude of the fit; and w_1_, w_2_, and w_3_ are widths for rising, falling, and combined sigmoid functions. This distribution determined the statistical peak energy transfer efficiency, E_peak_, which was compared to the calculated energy transfer E_cal_ = 1/[1+(r/R_0_)^6^]s where r and R_0_ are the actual and Förster (when E = 50%) distances between the donor and acceptor fluorophores.

Förster distances, R_0_, were calculated using the sixth root of
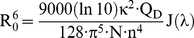
(2)where Q_D_ is the donor quantum yield, N is Avagadro's number, n is the index of refraction of the medium, and J(λ) is spectral overlap between the donor emission spectrum and the acceptor excitation spectrum as given by [48]

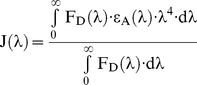
(3)where F_D_(λ) is the donor fluorescence emission as a function of wavelength, ε_A_(λ) is the acceptor molar extinction coefficient as a function of wavelength, and λ is the wavelength.

κ^2^ is the donor emission transition dipole-acceptor absorption transition dipole orientation factor with respect to the angle between the dipoles, and the angles between these dipoles and the vector joining them. Here, κ^2^ was assumed to be 2/3 as expected for freely rotating dipoles [48]. When the sixth root is used to calculate the actual distance between donor and acceptor pairs, variation from κ^2^ = 2/3 to 4 (for head-to-tail parallel transition dipoles) can be in error by no more than 35%. In order for our FRET experiments to be more in line with κ^2^ = 2/3, BMP ligand was not added to cells so that the binding between NRAGE-XIAP and corresponding dipole-dipole orientations of the donor and acceptor fluorophores would be more representative of freely rotating dipoles without influences from other BMP non-canonical members nor the plasma membrane. Therefore at worst case, 35% error would not change our results in assessing for FRET.

### Western blotting

For detection of phosphorylated IKK, HEK293 cells were seeded in 6-well plates and transfected at about 80% confluency with GeneJuice (EMD/Biosciences) per manufacturer's instructions. For detection of NRAGE repeat domain mutations F10R3 to F13R3, P19 cells were seeded in 6-well plates and transfected at about 80% confluency with Lipofectamine 2000 (Invitrogen) per manufacturer's instructions. 48 hours post-transfection, all cells were placed on ice and rinsed 3 times with ice cold PBS followed by lysing with RIPA buffer (150 mM NaCl, 10 mM Tris pH 7.2, 0.1% SDS, 1% Triton X-100, 1% deoxycholate, and 5 mM EDTA) containing protease (Calbiochem/EMD) and phosphatase inhibitors (Sigma). Cell lysates were centrifuged at 10,000 *g* for 10 minutes at 4°C and the supernatants were collected as total cell lyses. Protein concentrations were obtained by BCA assay (Pierce) per manufacturer's instructions. Lysates were loaded evenly by western blot for 8% or 10% SDS-PAGE electrophoresis. Protein was transferred to Hybond nitrocellulose membranes (Amersham), and blocked in either 5% non-fat dried milk or 5% BSA in washing buffer (1× TBS with 0.1% Tween-20). Membranes were probed with anti-phospho-IKK-α/β (Cell Signaling) or anti-EGFP antibody (Santa Cruz) followed by HRP conjugation with IgG (Bio-Rad). SuperSignal chemiluminescent substrate (Pierce) was used to detect HRP on HyBlot CL (Denville Scientific) or HyperFilm (Amersham) autoradiography film.

For western blotting and immunoprecipitation after NRAGE peptide treatment, HEK293 or P19 cells were seeded in 6-well plates on Day 0. On Day 1, 10 nM NRAGE peptide was delivered to cells using 6 µM EndoPorter reagent (Gene Tools). On Day 2, cells were serum starved for 4 hours before being treated with 10 ng/ml of BMP-4 (R & D Systems) for 1 hour and then lysed with either RIPA buffer for IKK or NP-40 lysis buffer (150 mM NaCl, 20 mM Tris pH 7.5, 1% NP-40, and 5 mM EDTA) for immunoprecitation containing protease and phosphatase inhibitors. Lysates were normalized by BCA protein assay (Pierce). HEK293 total cell lysates (TCL) were used for IKK and P19 cell immunoprecipitates were done with α-NRAGE antibody (Santa Cruz sc-14398) (IPs) detection of endogenous members. TCLs and IPs were loaded evenly for 10% SDS-PAGE electrophoresis, transferred to nitrocellulose membranes (Amersham), and blocked in either 5% nonfat dried milk or 5% BSA and washing buffer (1× TBS with 0.1% Tween-20). Membranes were probed with anti-XIAP (Cell Signaling), anti-TAK1 (Upstate), and anti-NRAGE (Santa Cruz sc-14400), and anti-phospho-IKK-α/β (Cell Signaling). SuperSignal chemiluminescent substrate (Pierce) was used to detect HRP on HyBlot CL (Denville Scientific ) autoradiography film.

For western blots of NRAGE peptide-YFP, P19 cells were seeded in 6 cm dishes and transfected with NRAGE peptide-YFP or YFP control with Lipofectamine 2000 per manufacturer's instructions (Day 0). On Day 1, NRAGE peptide-YFP, YFP control, or untransfected cells were seeded in 6-well plates in two sets at 250,000 cells per well. On Day 2, all medium was refreshed with one set receiving 1 µM retinoic acid and 10 ng/ml BMP-4 in the medium. On Day 3, all wells were lysed with NP-40 buffer containing protease and phosphatase inhibitors and total cell lyses were collected. TCLs were loaded evenly for western blotting as described above. Blots were probed with anti-caspase-3 (full length and cleaved; Cell Signaling), cytochrome c (Cell Signaling), and β-actin (Sigma) for loading control. Band intensity for cytochrome c was determined by ImageJ software revision 1.42n (National Institutes of Health) on an unadjusted ‘tif’ image with background subtracted, normalized to untreated wild-type P19 cells, and plotted by Microcal Origin (Microcal Software).

### NRAGE peptide design and manufacture

The NRAGE repeat domain was examined by hexameric amino acids in their inherent order and four similar hexapeptide repeat sequences – PPARQT, PPGWQS, PPDWPL, and PVAWQN – were determined. From this, a final consensus sequence of PPAWQT was reached (Fig. 6A). A 24-mer peptide with four repeats of the final consensus sequence (n-PPAWQTPPAWQTPPAWQTPPAWQT-c) was synthesized, cleaved, and lyophilized by Anaspec, Inc. (Fremont, CA). This sequence has a net neutral charge, a hydropathy index of −26.0, and four regions of polarity located at the QT dipeptides. All *in vitro* and explant experiments with the NRAGE peptide were conducted using unpurified peptide.

### NRAGE peptide DNA cloning

Nucleotide selection for the four amino acid consensus sequence repeats of the NRAGE peptide was intentionally selected for unique coding from end to end, includes a Xho I restriction enzyme site upstream to the transcription start site, and a Kozak sequence (Figure 6B). Both the sense and antisense strands were synthesized from Ultramer Oligo primers (Integrated DNA Technologies). See Table 2 for primer sequences. PCR cycles were 1 minute incubation at 94°C; 30 cycles of 30 seconds denature at 94°C, 30 seconds anneal at 62°C, 30 seconds extension at 65°C; 5 minutes final extension at 65°C, hold at 4°C using HotMaster Mix (Eppendorf). The PCR product was cloned into pGEM-T Easy vector (Promega) and excised using Xho I and EcoRI restriction enzymes (New England Biolabs). NRAGE peptide DNA was then cloned into pECFP-N1 and pEYFP-N1 (Clontech) vectors so that the 3′ end of the NRAGE peptide DNA was fused to the 5′ end of the tags with respect to the sense strand. NRAGE peptide DNA-CFP and -YFP were verified by sequencing and by exhibiting respective CFP and YFP fluorescence in transfected P19 cells using the Axiovert 200 micrscope (Carl Zeiss) and the CFP and YFP filter sets.

**Table 2:** NRAGE Peptide DNA Primers.

Sense	5′- CTCGAGCCCGCCACCATGGTGCCCCCAGCAT GGCAAACACCCCCTGCCTGGCAGACCCCGCCA GCGTGGCAAACGCCGCCTGCTTGGCAGACT -3′
Antisense	5′- GGCAGGGGGTGTTTGCCATGCTGGGGGCACC ATGGTGGCGGGCTCGAGAGTCTGCCAAGCAG GCGGCGTTTGCCACGCTGGCGGGGTCTGCCA -3′

### Apoptosis Array

NRAGE peptide-YFP and YFP control stably-integrated P19 cell lines were established using G418 selection. Cells were grown to confluency, lysed with NP-40 lysis buffer containing protease and phosphatase inhibitors, and concentrations determined by BCA assay (Pierce). 400 µg each of NRAGE peptide-YFP and YFP P19 cells were incubated on human apoptosis antibody nitrocellulose arrays (R & D Systems) per manufacturer's protocol and developed by HRP (Pierce) on HyperFilm (Amersham) autoradiography film. Antibodies were dotted in duplicate. Arrays were stripped with 0.2 M glycine pH 2.5, reincubated with NRAGE peptide-YFP and YFP control semi-stable P19 cell lysates after the cells were incubated with 1 µM retinoic acid and 10 ng/ml BMP-4 for 24 hours and developed by HRP on Hyperfilm.

### Apoptosis Assay

Cells were trypsinized and spun at 1200 rpm for 3 minutes to pellet. Cells were counted and diluted to 1 million per ml in antibody binding buffer included in the apoptosis detection kit (BD Biosciences). For controls, 100 µl of cells were placed in 5 ml culture tubes with 5 µl of either Annexin V-PE or 7AAD or both and incubated in the dark for at least 20 minutes. 250 µl of sample cells were incubated with 15 µl of each apoptosis marker for at least 20 minutes in the dark. Upon completion of the incubation period, cells were diluted in 400 µl (control) or 750 µl (sample) of binding buffer and analyzed by flow cytometry using the Becton Dickinson FASCCalibur flow cytometer and Cell Quest software version 3.3. GFP+ cells were gated and the incorporation of Annexin V-PE and 7AAD by the cells read. Gates were determined through the use of untransfected and unstained cells GFP and PE were excited by the Argon 488 nm laser and GFP read on the FL1 channel and PE on the FL2 channel.

### Proliferation assay

P19 cells were transfected as described above then on the morning of day 2 (24 hours post transfection) pulsed with 10 µg/mL BrdU for 2 hours. Cells were detached from the plate using 10 mM EDTA for 2 minutes at room temperature. 1 mL of media was added and the cells triturated off the plate into 15 mL conical tubes. Cells were spun at 400 rpm for 10 minutes to pellet. Media was removed and cells resuspended in 0.5% BSA in PBS (wash buffer) at a concentration of 1 million per 100 µL as recommended by BD Biosciences. Cells were pelleted again and fixed in ice cold 70% ethanol for 10 minutes. The fixation solution was diluted with 500 µL wash buffer and cells pelleted. Cells were then treated with 100 µL of 2 M HCl for 10 minutes to release the DNA from histones. Centrifugation at 400 rpm was used to pellet the cells and clear the acid; residual acid was neutralized with 0.2 M sodium borate for 3 minutes. Cells were washed again, pelletted, and resuspended in wash buffer plus 0.5% goat serum. A 1∶50 dilution of BrdU-Alexa647 and GFP-Alexa488 antibodies (Molecular Probes) were added and incubated for 20 minutes at room temperature in the dark. The cells were then washed twice to remove residual antibody, and analyzed by flow cytometry.

### Statistics

Sample size for FRET experiments was based on a one-sided *t* test with a level of significance of 0.05, a power level of 0.99, and one standard deviation [49]. *P* values were determined by single factor ANOVA performed in Excel (Microsoft,). Averages for E_peak_ and standard deviations were calculated on an Excel (Microsoft) worksheet and graphed using Origin (Microcal) software.

## Supporting Information

Figure S1 **An example of FRET analysisanalyses of NRAGE F4R2-EGFP:XIAP-DsRed and NRAGE F6R2-EGFP:XIAP-DsRed.** (A) Confocal false-color and pseudo-colorpseudocolor intensity images of NRAGE F4R2-EGFP show enhanced EGFP fluorescence after photo bleachingphotobleaching acceptor DsRed molecules identifying XIAP whereas NRAGE F6R2-EGFP images have hardly any increased fluorescence after DsRed photo bleachingphotobleaching (C). (B) Peak energy transfer efficiency is considerably higher for NRAGE F4R2-EGFP and XIAP-DsRed than for NRAGE F6R2-EGFP and XIAP-DsRed (D).
(EPS)

Figure S2 **ECFP:EYFP FRET recapitulates EGFP:DsRed FRET in which there is likely a direct interaction between NRAGE F4R2 and XIAP but not for NRAGE F6R2 and XIAP.** (A) Examples of confocal false-color and pseudo-colorpseudocolor intensity images of ECFP-NRAGE F4R2 show enhanced ECFP fluorescence after photo bleachingphotobleaching acceptor EYFP molecules identifying XIAP whereas ECFP-NRAGE F6R2 images have hardly any increased fluorescence after EYFP photo bleachingphotobleaching (C). (B) Corresponding peak energy transfer efficiency is considerably higher for ECFP-NRAGE F4R2 and EYFP-XIAP than for ECFP-NRAGE F6R2 and EYFP-XIAP (D).
(EPS)
